# Acute Adult‐Onset Still Disease Presenting as Fever of Unknown Origin and Severe Myalgia in a Resource‐Limited Tertiary Hospital in Brazil

**DOI:** 10.1155/carm/6925896

**Published:** 2026-07-29

**Authors:** Breno Bopp Antonello, Cainã Gonçalves Rodrigues, Giovanna Giovacchini dos Santos, Laura Grespan Dill, Paulo Victor Zattar Ribeiro

**Affiliations:** ^1^ Faculty of Medicine, Franciscan University, Santa Maria, Rio Grande do Sul, Brazil, franciscan.edu; ^2^ Faculty of Medicine, Federal University of Ceará, Fortaleza, Ceará, Brazil, ufc.br; ^3^ Faculty of Medicine of ABC, Santo André, São Paulo, Brazil; ^4^ Faculty of Medicine, Federal University of Santa Maria, Santa Maria, Rio Grande do Sul, Brazil, ufsm.br; ^5^ Department of Medical Genetics, Ribeirão Preto Medical School, University of São Paulo, Ribeirão Preto, São Paulo, Brazil, usp.br

## Abstract

This case highlights the diagnostic challenges of managing a patient who initially presented with a fever of unknown source and subsequently met the criteria for fever of unknown origin (FUO) after more than 3 weeks of persistent fever despite an extensive diagnostic workup in a resource‐limited tertiary hospital within Brazil’s public health system. A man in his sixth decade presented with prolonged high‐grade fever and severe lower‐limb myalgia, initially raising suspicion for leptospirosis due to rural exposure and occupational history. Extensive evaluation—including blood cultures, serologies, and imaging screening—revealed no infectious or malignant cause. Persistent neutrophilic leukocytosis, marked hyperferritinemia, and elevated inflammatory markers suggested a systemic autoinflammatory disorder. A trial of low‐dose prednisone produced partial improvement, and rheumatology consultation supported the diagnosis of adult‐onset Still disease (AOSD) based on Yamaguchi criteria. Corticosteroid escalation resulted in the rapid resolution of fever and myalgia. This case underscores the importance of structured reasoning, systematic exclusion of common causes, and careful attention to laboratory patterns for timely diagnosis in resource‐limited settings.

## 1. Background

This case report describes a rare disease presenting in a low‐complexity tertiary hospital within the Unified Health System (SUS) in southern Brazil. The institution provides all care, free of charge, and functions with limited diagnostic resources. The diagnostic process was particularly challenging because several advanced investigations were not available, making clinical reasoning and the systematic exclusion of alternative diagnoses essential. This case represents the investigation of a patient who initially presented with a fever of unknown source and subsequently met the criteria for fever of unknown origin (FUO) in a previously healthy adult and illustrates the diagnostic challenges faced in resource‐limited settings. It highlights the importance of systematic clinical reasoning, broad differential diagnosis, and stepwise exclusion of infectious, malignant, and autoimmune etiologies. By presenting this case, we aim to guide other clinicians on how to approach undifferentiated febrile illnesses, emphasizing the importance of careful history‐taking, thorough physical examination, judicious use of laboratory and imaging studies, and early consideration of rare or atypical conditions when common diagnoses are excluded.

## 2. Case Presentation

A man in his 60s from a rural area in the southern part of Brazil presented with a 4‐day history of high‐grade fever (peaking at 39.8°C/103, 64°F), diffuse headache, and intense bilateral lower‐limb myalgia and arthralgia severe enough to limit ambulation. The pain originated in the thighs and radiated to the calves, with a reported intensity of 8/10. He also noted chills and the appearance of small nodules on the scalp. Earlier in the week, he had experienced transient cervical lymphadenopathy that resolved spontaneously. He denied weakness, rash, sore throat, nausea, diarrhea, cough, dyspnea, dysuria, confusion, or other symptoms. Oral intake remained adequate, and he reported no contact with individuals with similar complaints. He initially sought evaluation at a primary health unit and was subsequently admitted to the hospital on the evening of the next day, accompanied by his wife. At the time of admission, he continued to report severe lower‐limb pain without additional symptoms. He also recalled possible contact with rats while cleaning a shed in the preceding weeks.

He had worked as a butcher and livestock farmer before becoming a bakery employee. His medical history included hypertension controlled with Losartan. He was a former smoker and reported an allergy to ibuprofen. He had no prior autoimmune or rheumatologic disease nor family history of such diseases. On admission, the patient appeared in good general condition. Physical examination was unremarkable except for moist but pale mucous membranes and tenderness of the calves and lower legs.

Initial laboratory evaluation demonstrated a white‐cell count of 16,100 per cubic millimeter, with 6% bands and 73% segmented neutrophils, hemoglobin of 15.5 g per deciliter, and normal platelet count, renal function, and creatine phosphokinase levels. Given the patient’s rural background and history of animal exposure, leptospirosis was suspected, and empiric ceftriaxone (1 g every 12 h) was initiated.

On follow‐up, the patient was observed in his hospital bed, seated, cooperative, and communicative. He reported intermittent lower‐limb pain overnight that worsened as body temperature rose, which was well controlled with analgesics in the first days. He was unable to clearly differentiate whether the pain originated from the muscles or the joints; however, he consistently reported bilateral knee pain. The patient did not report any skin rash, nor was one observed in the physical examination. Urinary output remained regular, and oral intake of food and fluids was satisfactory. Notably, he had a healed cut on his left hand from woodworking in the preceding weeks, raising the consideration of possible hematogenous infections. He also denied any new or additional complaints but reported an important unintentional weight loss of 10 kg/22 lb in the last months, considering the suspicion of malignancy and neoplasia.

After 8 days of ceftriaxone, the patient continued to experience predominantly nocturnal fever, and his leg pain worsened without response to analgesics. A broad infectious workup—including serologic testing for leptospirosis, dengue, brucellosis, cytomegalovirus, HIV‐1 PCR, and Epstein–Barr virus—was negative. Imaging of the chest, abdomen, spine, and legs revealed no infectious or neoplastic focus, and echocardiography showed no evidence of valvular vegetations; therefore, infective endocarditis was considered unlikely. Serum protein electrophoresis showed no monoclonal peak.

Abdominal CT revealed some increased density of the perirenal fat, raising the possibility of renal infection. Systemic lupus erythematosus was also considered; however, renal function remained normal, and no involvement of other organs was evident. Prostatitis was considered a differential diagnosis, but PSA was normal, and no urinary tract symptoms were present. A contrast‐enhanced abdominal CT was obtained to evaluate for vasculitis, as tissue biopsy was not feasible at this hospital, but no significant abnormalities were identified.

Considering the patient’s recent cut on his hand and the potential masking of blood cultures due to ongoing antibiotic therapy, as well as the possibility of a Gram‐positive infection such as *Staphylococcus aureus*, vancomycin was added on hospital day 9 to cover this and other possible resistant organisms. The patient reported mild symptomatic improvement the following day, with partial reduction in fever; however, the febrile episodes soon recurred.

Despite transient symptomatic relief, the patient continued to exhibit a pronounced inflammatory response. Laboratory evaluation demonstrated progressive leukocytosis, increasing from 16,100/mm^3^ at admission to a peak of 25,300/mm^3^ during hospitalization, accompanied by neutrophilia and a left shift. The proportion of segmented neutrophils reached 80.6% (17,732/mm^3^) at its highest recorded value. Creatine kinase remained low at 28 U/L. Serologic testing for viral hepatitis (HBsAg and HCV), HIV, and syphilis (VDRL and Treponema pallidum) was negative. Serial blood cultures remained sterile throughout hospitalization.

After an extensive evaluation for FUO, with no infectious focus identified, additional rheumatologic and autoimmune investigations were pursued, raising suspicion for a systemic autoinflammatory disorder. Inflammatory parameters were markedly elevated, with an erythrocyte sedimentation rate of 95 mm per hour and serum ferritin of 4234 ng per milliliter. Autoimmune screening, including antinuclear antibodies (ANA) and rheumatoid factor (RF), was negative, whereas complement levels were reduced (C3, 60 mg/dL; C4, 4 mg/dL).

After an extensive investigation failed to identify an infectious or malignant etiology, an autoinflammatory disorder was suspected. Given the unavailability of a rheumatologist at the time and the presence of persistent evening fevers exceeding 38°C–39°C/100, 4‐102, 2 F unresponsive to antipyretics, a therapeutic trial of low‐dose prednisone (10 mg daily) was initiated, targeting a possible autoimmune or rheumatologic process. This led to partial symptomatic improvement. Subsequent rheumatology consultation supported a diagnosis of adult‐onset Still disease (AOSD) based on Yamaguchi criteria, including the combination of three major criteria: fever ≥ 39°C (102.2°F) lasting ≥ 1 week, leukocytosis ≥ 10,000/μL with ≥ 80% granulocytes, arthralgia lasting ≥ 2 weeks, and two minor criteria: a negative ANA and RF and a history of lymphadenopathy. After deworming, prednisone was escalated to 60 mg daily, resulting in complete resolution of fever and leg pain within 48 h.

He was discharged after 18 days of hospitalization on oral corticosteroids, with outpatient follow‐up with a rheumatologist. Five days later, methotrexate was introduced as a steroid‐sparing agent. At follow‐up, he remained afebrile and asymptomatic with the final diagnosis of AOSD based on Yamaguchi criteria.

## 3. Differential Diagnosis

### 3.1. FUO

Patients presenting with classic FUO often experience prolonged fevers, and many may initially undergo outpatient evaluation—either as a first step or sequentially—without immediate hospitalization [1]. A meticulous history and thorough physical examination are crucial, as the information gathered typically guides the diagnostic workup and helps narrow potential etiologies [1].

The initial diagnostic evaluation for FUO generally includes a standardized panel of tests, with additional investigations tailored to specific clinical clues identified in the history or examination [1, 2]. The minimum initial workup typically consists of a complete blood count with differential and peripheral smear, a comprehensive metabolic panel including calcium, liver function tests, and two sets of blood cultures from separate sites obtained prior to antibiotic therapy [1]. Additional baseline studies include urinalysis with reflex urine culture, HIV antigen/antibody testing, ANA, RF, heterophil antibody, or Epstein–Barr virus testing in younger adults, erythrocyte sedimentation rate, C‐reactive protein, ferritin, and, in endemic areas, thick and thin blood smears for malaria [1, 2]. Imaging with CT of the chest, abdomen, and pelvis is also recommended to detect potential infectious, inflammatory, or malignant foci [1, 2].

Subsequent testing, including tissue biopsy, may be indicated if clinical data suggest a specific diagnosis that can be confirmed histologically [2]. Empiric antibiotics are generally reserved for patients with suspected life‐threatening infections, while empiric glucocorticoids are avoided unless the patient is severely ill or a steroid‐responsive condition—such as giant cell arteritis—is strongly suspected [2].

Despite thorough evaluation, up to half of patients with classic FUO may remain without a definitive diagnosis. Fortunately, most undiagnosed adults have a favorable prognosis [2]. This approach underscores the importance of structured, stepwise assessment, careful clinical reasoning, and judicious use of empiric therapies to optimize patient outcomes while avoiding unnecessary interventions [1, 2].

### 3.2. Leptospirosis

Leptospirosis is a worldwide zoonotic infection caused by pathogenic *Leptospira* species and is particularly prevalent in tropical regions [3, 4]. Humans typically acquire the infection through contact with water or soil contaminated with the urine of infected animals, especially rodents [3, 4]. Risk factors include rural exposure, farming activities, livestock handling, and occupational contact with contaminated environments [3, 4].

Clinical manifestations range from mild febrile illness to severe, life‐threatening disease [3, 5]. The most common presentation is anicteric leptospirosis, characterized by abrupt‐onset fever, headache, and prominent myalgia, particularly involving the calves and lower back [3]. More severe forms may present with jaundice, acute kidney injury, pulmonary hemorrhage, and multiorgan dysfunction [3].

Leptospirosis was initially considered a leading diagnostic hypothesis because of the patient’s rural background, occupational exposure to livestock, possible recent contact with rodents, and prominent calf myalgia, a classic feature of the disease. However, several clinical and laboratory findings argued against this diagnosis. The patient did not develop the characteristic biphasic course of anicteric leptospirosis, nor did he exhibit conjunctival suffusion, jaundice, renal dysfunction, thrombocytopenia, or pulmonary involvement. Despite severe lower‐limb pain, creatine kinase levels remained normal, making significant leptospiral myositis less likely. Furthermore, the persistent quotidian fever pattern, progressive neutrophilic leukocytosis, marked hyperferritinemia, and sustained inflammatory response were not typical of leptospirosis. Most importantly, serologic testing for leptospirosis was negative. Taken together, the absence of characteristic clinical manifestations, the atypical disease course, and the negative diagnostic testing made leptospirosis an unlikely explanation for the patient’s presentation, leading to its exclusion from the differential diagnosis.

### 3.3. Dengue

Dengue virus infection is a mosquito‐borne viral illness that can present across a spectrum of severity, ranging from asymptomatic infection to severe, life‐threatening disease [6, 7]. Clinically, dengue often begins with a sudden onset of high fever accompanied by headache, retro‐orbital pain, myalgia, arthralgia, nausea, and vomiting [6]. A tourniquet test—performed by inflating a blood pressure cuff on the arm to midway between systolic and diastolic pressures for five minutes—may reveal petechiae within one to 2 minutes after cuff deflation [6]. Laboratory findings commonly include leukopenia, thrombocytopenia, and elevated aminotransferases [6, 7]. Diagnosis of dengue is primarily clinical, based on the presence of typical manifestations—fever, headache, retro‐orbital pain, myalgia, arthralgia, rash, and hemorrhagic signs—combined with recent exposure to a dengue‐endemic area [6, 7].

Although dengue is highly prevalent in tropical countries and was considered in the differential diagnosis, our patient did not fulfill the diagnostic criteria for dengue infection. He lacked the typical clinical manifestations of the disease, including thrombocytopenia, hemorrhagic features, and the characteristic biphasic clinical course. Instead, he presented with a persistent quotidian fever pattern without an afebrile interval. Furthermore, the progressive clinical evolution and systemic inflammatory manifestations were not consistent with dengue fever. In addition, dengue NS1 antigen testing was negative, providing further evidence against acute dengue infection. Taken together, these findings made dengue an unlikely explanation for the patient’s presentation, and the diagnosis was ultimately excluded.

## 4. Discussion

FUO remains one of the most challenging diagnostic entities in internal medicine, particularly in resource‐limited settings. This case illustrates how an apparently acute infectious presentation—high‐grade fever, intense calf myalgia, and rural exposure—may ultimately reflect a systemic autoinflammatory disorder rather than infection. The patient’s earliest features, including neutrophilic leukocytosis and a left shift, initially suggested bacterial disease, yet the systematic exclusion of leptospirosis and other common tropical infections prompted reconsideration of the diagnostic pathway, as this patient had not fulfilled all criteria for leptospirosis or other infectious diseases. This case reinforces an essential teaching point: not all leukocytosis with neutrophilia and left shift indicates infection, and clinicians must remain attentive to inflammatory and autoinflammatory etiologies [8, 9].

AOSD results from dysregulated innate immunity leading to excessive cytokine production—particularly interleukin‐1 (IL‐1), IL‐6, and IL‐18—producing high spiking fevers, systemic inflammation, and, in some cases, macrophage activation syndrome (MAS) [9]. The Yamaguchi criteria (Table 1) remain the most widely applied diagnostic framework, requiring exclusion of infections, malignancy, and autoimmune disease [10]. Diagnosis for AOSD requires five criteria, with at least two major criteria. Our patient fulfilled three major criteria (fever ≥ 39 °C lasting ≥ 1 week, leukocytosis ≥ 10,000/μL with ≥ 80% granulocytes, and arthralgia lasting ≥ 2 weeks) and two minor criteria (negative ANA/RF and a history of cervical lymphadenopathy), thereby meeting the Yamaguchi classification criteria for AOSD after alternative infectious, malignant, and rheumatic diagnoses had been excluded. Marked hyperferritinemia further supported the diagnosis [8, 10, 11].

**TABLE 1:** Yamaguchi criteria.

Category	Criteria
Diagnostic Requirement	Diagnosis requires five criteria, with at least two major criteria. The presence of infection, malignancy, or other rheumatic disorders that mimic AOSD precludes the diagnosis.

Major Criteria	• Fever ≥ 39 °C (102.2 °F) lasting ≥ 1 week • Arthralgia/arthritis lasting ≥ 2 weeks; • Nonpruritic salmon‐colored rash, macular or maculopapular, typically on trunk or extremities during febrile episodes; • Leukocytosis ≥ 10,000/μL with ≥ 80% granulocytes.

Minor Criteria	• Sore throat; • Lymphadenopathy; • Hepatomegaly or splenomegaly; • Abnormal liver function tests; • Negative ANA and RF.

Hyperferritinemia is an important diagnostic clue. Although ferritin levels can rise in the context of infection or malignancy, markedly elevated values—as seen in this patient—are particularly suggestive of AOSD [11, 12]. Ferritin is not merely an acute‐phase reactant; it plays a direct pathogenic role by promoting macrophage activation and iron‐mediated oxidative stress [9]. Early recognition of this laboratory signature is therefore critical. In this case, however, interpretation of the marked ferritin elevation was delayed because infectious and neoplastic workups—necessary to exclude more common etiologies—were not promptly available and required several days to complete. Given initial concerns for potential sepsis, the patient was managed as having an infectious process. This approach ultimately proved suboptimal, and during the first week of evaluation, the possibility of an underlying rheumatologic disease was prematurely deprioritized when it should have remained part of the FUO workup [8].

Nevertheless, in accordance with the FUO evaluation guidelines, our patient underwent a structured diagnostic approach, which included a detailed history and examination, broad infectious and autoimmune laboratory testing, and cross‐sectional imaging [8]. This systematic strategy prevents premature anchoring and reduces unnecessary empiric therapy. Therefore, given the absence of treatable infection and the progressive inflammatory profile, a cautious trial of low‐dose corticosteroid therapy was reasonable [8, 9]. Although the dramatic response to prednisone escalation to 60 mg supported the diagnosis of AOSD, it was not diagnostic, as corticosteroid responsiveness is not specific to this condition. Nonetheless, symptom resolution occurred rapidly after treatment initiation (Table 2).

**TABLE 2:** Summarized complete patient history.

Days	Symptoms
1	• Onset of high‐grade fever (39 °C) and transient cervical lymphadenopathy
2	• Development of severe lower‐limb myalgia and arthralgia
3	• Progressive worsening of myalgia and arthralgia with persistent fever
4	• Sought initial medical evaluation; leptospirosis considered the leading diagnostic hypothesis.
5 (Hospital Day 0)	• Hospital admission; cervical lymphadenopathy no longer evident on examination; • Empiric ceftriaxone (1 g every 12 h) initiated because of suspected leptospirosis.

6–13 (Hospital Days 1–8)	• Persistent high‐grade evening fevers, typically beginning around 5:00 PM and resolving overnight; • Broad differential diagnostic evaluation pursued because of persistent nocturnal fever; • Fever became increasingly refractory to antipyretic therapy; • Progressive inflammatory response, with worsening leukocytosis and left shift; • No new symptoms reported; lower‐limb myalgia and arthralgia remained the predominant complaints.

14 (Hospital Day 9)	• Empiric vancomycin therapy added to broaden antimicrobial coverage.
15–20 (Hospital Days 10–14)	• Extensive evaluation performed to exclude infectious and malignant etiologies; • No evidence of infection or malignancy identified; • Autoinflammatory and autoimmune disorders increasingly considered; • Low‐dose prednisone (10 mg/day) initiated because of persistent high‐grade nocturnal fever.

21 (Hospital Day 15)	• Rheumatology consultation and review of ferritin, ANA, RF, C3, and C4 results; • AOSD emerged as the leading diagnostic hypothesis.

22 (Hospital Day 17)	• Empiric deworming therapy initiated • Prednisone 60 mg/day planned; • Formal diagnosis of fever of unknown origin (FUO) established after 21 days of persistent fever.

23 (Hospital Day 18)	• Partial symptomatic improvement following low‐dose corticosteroid therapy; • Outpatient rheumatology follow‐up arranged; • Patient discharged with planned outpatient follow‐up.

24‐26 (Outpatient)	• Fever persisted despite gradual improvement in lower‐limb myalgia and arthralgia.
27 (Outpatient)	• Prednisone 60 mg/day initiated
28 (Outpatient)	• Rapid clinical improvement • Last documented febrile episode

29 (Outpatient)	• Afebrile; lower‐limb myalgia and arthralgia nearly completely resolved
30 (Outpatient)	• Rheumatology follow‐up consultation; near‐complete resolution of symptoms reported; • Patient kept accompaniment with the rheumatologist to treat accordingly; • Methotrexate initiated as a steroid‐sparing agent; • Diagnosis of AOSD confirmed.

Similar presentations of AOSD without the characteristic rash have been reported, particularly among older adults, and can contribute to delays in diagnosis [9, 13]. However, cases like this one—lacking both rash, persistent, or fluctuant lymphadenopathy and a story of pharyngitis—are less commonly described and may often exhibit an atypical, rapidly progressive onset rather than the more chronic course with weeks of arthralgia [13]. Also, the presence of low complement levels and significant weight loss represented atypical findings and contributed to the diagnostic uncertainty. Although hypocomplementemia is not classically associated with AOSD, rare cases have been reported in the literature, including a reported case of AOSD associated with hypocomplementemia. [14]. Furthermore, despite the low C3 and C4 levels, the patient lacked other clinical or serological features suggestive of systemic lupus erythematosus or another immune‐complex–mediated disease, including a negative ANA, preserved renal function, and absence of multisystem involvement. Significant weight loss may also occur in prolonged inflammatory diseases and has been described in patients with AOSD presenting as a FUO.

Several important alternative diagnoses were carefully considered during the diagnostic evaluation. Malignancy was initially suspected because of the patient’s unintentional weight loss and prolonged fever. However, extensive imaging failed to identify a solid tumor, lymphadenopathy, organomegaly, or other evidence of neoplastic disease, and serum protein electrophoresis revealed no monoclonal gammopathy. Infective endocarditis and other occult infections were also considered because of the persistent fever and neutrophilic leukocytosis. Nevertheless, serial blood cultures remained negative, echocardiography showed no evidence of valvular vegetations, broad infectious serologies were unrevealing, and no infectious focus was identified on repeated imaging studies. Systemic lupus erythematosus was considered because of the low complement levels; however, the absence of characteristic clinical manifestations, normal renal function, negative ANA testing, and lack of multisystem involvement made this diagnosis unlikely. Similarly, vasculitis was considered, but contrast‐enhanced imaging demonstrated no vascular abnormalities, and there was no evidence of organ ischemia, glomerulonephritis, pulmonary involvement, or other features suggestive of systemic vasculitis. MAS was also considered because of the marked hyperferritinemia. However, the patient did not develop cytopenias, liver dysfunction, coagulopathy, hypertriglyceridemia, splenomegaly, or other characteristic features of MAS. In contrast, he exhibited persistent neutrophilic leukocytosis and maintained normal platelet counts throughout hospitalization. Therefore, the available clinical and laboratory findings did not support a diagnosis of MAS.

Q fever, caused by Coxiella burnetii, deserves consideration as a potential differential diagnosis in patients presenting with FUO and a history of livestock exposure [15]. Although this diagnosis was not initially pursued during the patient’s hospitalization, his previous occupation as a livestock farmer represents a recognized epidemiological risk factor. Nevertheless, the absence of clinical manifestations commonly associated with acute Q fever, such as atypical pneumonia, hepatitis, or significant constitutional and gastrointestinal symptoms, made this diagnosis less likely. Therefore, although Q fever was not specifically investigated, the overall clinical presentation and subsequent evolution toward AOSD made this diagnosis unlikely.

This case highlights the need for clinical vigilance regarding autoinflammatory disorders in FUO—especially when laboratory findings suggest intense systemic inflammation despite exhaustive negative infectious and neoplastic studies. Attention to patterns such as hyperferritinemia, neutrophilic leukocytosis, and steroid responsiveness can accelerate diagnosis of AOSD and optimize outcomes [8, 9, 11]. A rapid clinical response to corticosteroids is typical of AOSD, although steroid‐sparing IL‐1 inhibitors are increasingly recommended for refractory disease [8, 9]. Two weeks after discharge, our patient had achieved complete functional recovery and returned to normal activities, reinforcing the effectiveness of early, targeted therapy.

## 5. Patient’s Perspective

It all began on Friday. By Saturday, my symptoms had worsened, and on Sunday, I went out to pick up some items for a sales stand I run. Later that same day, the fever started. On Monday, I went to a local clinic and was admitted to the hospital. My condition continued to deteriorate, and there were days during hospitalization when I felt like I might not recover. It was extremely difficult—I had severe, intense leg pain and at one point needed to use a wheelchair to move around. However, after receiving the appropriate treatment and finally obtaining a diagnosis, I began to feel better and experienced a great sense of relief as my condition gradually improved.

## 6. Learning Points/Take Home Messages

• AOSD should be considered in adults presenting with FUO, myalgia or arthralgia, neutrophilic leukocytosis, and markedly elevated ferritin, particularly after infectious and malignant causes are excluded.
• Resource‐limited settings may hinder extensive diagnostic testing, making careful history, physical examination, and stepwise exclusion critical for diagnosis.
• Early recognition of AOSD and timely initiation of corticosteroid therapy can lead to rapid symptom resolution and prevent complications, including MAS.
• Hyperferritinemia and persistent systemic inflammation are important clues pointing toward autoinflammatory disorders when standard infectious and autoimmune workups are negative.

Not all leukocytosis with neutrophilia and left shift indicates infection. Persistent neutrophilic leukocytosis can result from systemic inflammatory disorders, malignancy, tissue necrosis, or stress responses. In a FUO, sterile cultures and the absence of a clear infectious focus should prompt consideration of autoinflammatory conditions such as AOSD.

## Author Contributions

Conceptualization: Breno Bopp Antonello. Methodology: Breno Bopp Antonello, Cainã Gonçalves Rodrigues, and Giovanna Giovacchini dos Santos. Writing–original draft: Breno Bopp Antonello, Cainã Gonçalves Rodrigues, Giovanna Giovacchini dos Santos, and Laura Grespan Dill. Writing–review and editing: Paulo Victor Zattar Ribeiro, Breno Bopp Antonello, Cainã Gonçalves Rodrigues, Giovanna Giovacchini dos Santos, and Laura Grespan Dill. Supervision: Paulo Victor Zattar Ribeiro.

All authors meet the criteria for authorship. Each author made substantial contributions to the conception and design of the study, data acquisition, analysis, or interpretation and drafted or critically revised the manuscript.

## Funding

This study received no external funding.

## Disclosure

This manuscript is original, has not been previously published, and is not under consideration for publication elsewhere. All authors have read, approved the final manuscript, and agreed to be accountable for all aspects of the work.

## Ethics Statement

This study was conducted in accordance with relevant ethical guidelines and regulations.

## Consent

Written informed consent was obtained from the patient for publication of this case report and any accompanying images. All procedures were performed in accordance with institutional ethical standards and the principles of the Declaration of Helsinki.

## Conflicts of Interest

The authors declare no conflicts of interest.

## Data Availability

No datasets were generated or analyzed during this study, as this is a case report.
